# News: Agencies

**Published:** 2011-12

## The Bill and Melinda Gates Foundation

### Bill Gates delivers long-awaited speech on the future of development finance to G20 leaders

Invited by the French President Mr Sarkozy to address the world leaders at the G20 Summit in Cannes, France, and share his views on the future of development finance, Mr Bill Gates has delivered one of the most awaited and important speeches this year. His messages were being very carefully analyzed, interpreted and debated throughout the world press, with the response to his contribution to the summit being overwhelmingly positive. Mr Jonathan Glennie, writing for The Guardian, summarized the three key elements of Mr Gates’ address to G20, saying that Mr Gates: “…emphasised the key features of this era of development: the steps forward in terms of development indicators, relative to previous decades. He calculated that, by focusing on good news rather than calamity, he would inspire world leaders to continued action. Gates gave great examples of change, and his intention was clear – to inspire people at a time of economic gloom. In this sense, he has moved from businessman to statesman.” Mr Glennie then added that Mr Gates “…underlined what he called the “paramount importance of innovation” – his calling card. Gates's support for innovation and knowledge transfer makes him a natural supporter of south-south style development co-operation, which emphasises mutual learning as much as financial transfers. His explanation of how triangular co-operation works will be one of the key things many western leaders – still mostly illiterate in the ways the south is helping itself – take from his speech”. Finally, Mr Glennie concluded that Mr Gates “…noted that some countries are emerging from aid dependence, which shifts focus firmly on to revenue mobilization.” Mr Glennie expressed his personal view that “…the legally binding transparency requirements for extractive industries are far more important for development than the transaction tax, that has won all the headlines”, because these requirements would generate resources and greater accountability in poor countries, which is the crucial link to institutional progress.

### Forbes declares Melinda Gates world's 6th most powerful woman

The most recent annual ranking of the 100 women in the world with the greatest clout and influence, which is regularly performed by the Forbes magazine, declared Ms Melinda Gates the 6th most powerful woman in the world. The co-founder and co-chair of the Bill & Melinda Gates Foundation has outranked Michelle Obama (ranked 8th), Lady Gaga (ranked 11th), Queen Elizabeth II (ranked 49th) and former House Speaker Nancy Pelosi (ranked 52nd).

### Novartis’ head of development to lead global health at The Gates Foundation

The Bill & Melinda Gates Foundation have announced that Dr Trevor Mundel has been named their president of the Global Health Program. Dr Mundel, currently working as global head of development for Novartis Pharma AG based in Basel, Switzerland, took his post in December. The move is expected to further strengthen an already strong co-operation between leading pharmaceutical companies and the world’s largest philanthropic organisation. The global health at The Gates Foundation has about US$ 1.5 billion (€ 1.2 billion) annual budget and it supports the development of new drugs, vaccines and diagnostic tools.

### PATH’s president to lead global development at The Gates Foundation

Chris Elias, President & CEO at PATH, an international nonprofit organization, will step down from his current position and join the Bill & Melinda Gates Foundation (BMGF) as President for Global Development in February 2012. Writing for the Centre for Global Development, Ms Nandini Oomman said that first reactions from many in global health were a surprised ones, as Mr Elias was widely recognized for his contributions to global health, and much less so to the global development. However, a more detailed analysis, according to Ms Oomman, points to the conclusion that this appointment may help bridge gaps between global health and global development and integrate the two. Integrating global health delivery into global development would result in much greater impact of global health activities, while linking the discovery and development of interventions to their delivery would fill the gap in translation that frequently exists. Ms Oomman concludes that this kind of cross-disciplinary appointments is a growing trend in a multidisciplinary world of global health, and it will be expected to have positive implications in integrating the field.

### The Gates Foundation to award US$ 35 million for innovative ideas in family health

The Bill & Melinda Gates Foundation announced that it will invest US$ 35 million (€ 27 million) in grants to “…expand the pipeline of groundbreaking ideas that can help women and children live more prosperous and healthy lives”. The funding was announced at the annual Grand Challenges Meeting in Delhi, India. It will fund two new Grand Challenges in Global Health grant programs through supporting research into innovative solutions for family health. Mr Chris Wilson, director of Global Health Discovery at the foundation, commented that “…there is a vital need for new and creative ideas to help mothers and children in the world’s poorest countries.”

## The Gavi Alliance

### GAVI chief on innovative finance mechanisms that led to success

Speaking for the Global Health Magazine, GAVI Alliance’s chief, Mr Seth Berkley, discussed innovative finance mechanisms that contributed to GAVI’s success. Innovative ways to deliver vaccines to children in low resource settings is at the heart of GAVI Alliance’s mission which brings together governments, international organizations such as UN’s bodies, vaccine manufacturers and other industry partners, civil society, large donors and philanthropists and private corporations. They share the common goal of “…saving lives and improving health by expanding access to immunization in developing countries”. As stated by Mr Berkley, “…innovative finance is designed to provide more money for health and more health for the money”.

### Three innovative finance products created by GAVI explained

According to Global Health Magazine’s interview with GAVI Alliance’s chief, Mr Seth Berkley, GAVI has created three innovative finance products that underlie its efficient operations. The first is “GAVI Matching Fund”, designed to raise US$ 260 million (€ 201 million) for immunization by the end of 2015. Under this program, the UK Department for International Development (DFID) and the Bill & Melinda Gates Foundation have pledged about US$ 130 million (€ 100 million) combined to match contributions from corporations, foundations and other organizations, their customers, employees and business partners. The second one is “International Finance Facility for Immunisation” (IFFIm), which uses long-term payment pledges from nine donor governments (and Brazil to join soon) to create and sell “vaccine bonds” in the capital markets and ensure cash flow for the organization to purchase vaccines. It was started in 2006 as the first ever aid-financing entity to attract legally binding commitments of up to 20 years. Vaccine bonds have proven popular with all investors who are looking for both a market-based return and a socially responsible investment. The third one is “Advance Market Commitment” (AMC). It funds newer and expensive vaccines through connecting donors, the World Bank, UNICEF, WHO and the vaccine industry to provide vaccines at significantly reduced prices. Through this mechanism, companies sign a legally binding agreement to provide the vaccines at a price that would be both affordable and sustainable for developing countries in the longer term. By shaping markets, GAVI helps to reduce the prices of vaccines over time.

### Praise for GAVI Alliance’s innovative approach to international development aid

During the UN General Assembly in New York in September 2011, both leaders of the governments' aid programmes in USA and the UK have recognised the GAVI Alliance as “…offering “game changing” lessons in the fight against global poverty”. Mr Raj Shah, who is the acting Head of USAID, and Mr Andrew Mitchell, presently UK’s Secretary of State for International Development, have both highlighted GAVI as a “…model global development partnership that is significantly helping advance the Millennium Development Goals (MDGs)”.

### Childhood pneumonia is now a dominant target of GAVI grants

Childhood pneumonia has been the leading cause of child deaths globally for many decades, but very few policy makers were aware of this only several years ago. The work by WHO/UNICEF’s Child Health Epidemiology Reference group (CHERG) drew attention to pneumonia as the leading killer of children. A number of subsequent activities, including an assessment of the burden of specific causes of pneumonia – such as *S. pneumoniae* and *H. influenzae* type B – made it apparent that the majority of the burden could be prevented through pneumococcal and Hib vaccination. This year, in the largest-ever approval of grants for vaccines announced by the GAVI alliance, almost two-thirds of the funds would be spent on pneumonia prevention. *Development Today* reported that, of US$ 1 billion (€ 0.7 billion) intended for spending, US$ 664 million (€ 512 million) would be spent on purchasing pneumococcal vaccine for 36 million children in 12 African countries that have applied for support.

### GAVI aims to introduce HPV and rubella vaccines in developing countries

According to Reuters, The GAVI Board announced in November that it “...will take the first steps towards the introduction of HPV and rubella vaccines in developing countries”. Commenting on the decision to support HPV vaccine introduction, GAVI said that “…if negotiations to secure a sustainable price from manufacturers are successful and countries can demonstrate their ability to deliver the vaccines, up to two million women and girls in nine countries could be protected from cervical cancer by 2015”. Moreover, the GAVI Alliance “…has agreed to fund the roll-out of vaccines against cervical cancer in developing countries, offering protection against a disease that kills one woman every two minutes”.

## The World Bank

### World Development Report 2012 focuses on gender equality

Globally, women's rights are improving, both in absolute terms and relative to men. The World Bank's report says that in high-income countries there's a consensus supporting legal rights and guarantees of equality for women. In many middle and low income countries more women are literate and their education level is getting nearer that acheved by men. Women's share in the global workforce is now up to 40%, and in agricultural workforce it is even higher – 43%. Encouragingly, more than half of the university students globally are now females. Ana Revenga, co-director of the World Bank's World Development Report 2012, said upon the release of the report earlier this year that “...in today's globalized world, countries that use the skills and talents of their women will have an advantage over those that don't”.

### UN chief sees investing in people as the way to overcome poverty

According to UN News, Secretary-General Ban Ki-moon cautioned that “...progress so far in the fight against poverty risked being reversed by a failure to put people at the centre of development policies and strategies aimed at economic recovery following the global financial crisis. In the name of fiscal austerity, we cannot cut back on common-sense investments in people”. Mr Ban sent this message in a speech which marked the International Day for the Eradication of Poverty. He also noted that “...too many people have been seized by the fear of losing their jobs, their ability to feed their families and access to health care. We can meet the challenges we face – the economic crisis, climate change, rising cost of food and energy, the effects of natural disasters. We can overcome them by putting people at the centre of our work”.

### An independent review panel urges Global Fund to reform

According to The Guardian, a report from an independent high-level panel found major flaws in both the governance and oversight of the Global Fund, in spite of its good overall performance and clear accomplishments. The review was chaired by former US Health Secretary Michael Leavitt and former President of Botswana Festus Mogae. The verdict of the panel was that the Global Fund must “change or wither”. The report followed a major crisis of confidence in the Fund, after the media reports at the end of 2010 implicated fraud and corruption among countries taking Global Fund money. It is now feared that this kind of conclusion could give donors the excuse to reduce or entirely discontinue their funding support to the Global Fund. This would be devastating for the efforts against HIV/AIDS, tuberculosis and malaria in low and middle income countries. In January this year, Germany had already suspended payments and there was talk of other nations also turning away.

### Eurodad warns that the majority of development aid is “boomerang aid”

A study released in September in Brussels by the European Network on Debt and Development (Eurodad), a network of 58 non-governmental organisations from 19 European countries, claimed that “...development aid is ineffective mostly because it is tied to contracts worth billions of dollars awarded to firms in developed countries, in a phenomenon called boomerang aid”. The study showed that more than two-thirds of all aid contracts were eventually awarded to companies in the wealthy countries. The study was deliberately released ahead of the Fourth High Level Forum in Busan, South Korea, bringing together the world’s governments and stakeholders in November 2011 to consider how to make aid more effective. Organisation for Economic Cooperation and Development (OECD) estimated the total development assistance in 2009 at US$ 128 billion (€ 99 billion). Bodo Ellmers of Eurodad, who prepared the report, said that “...most people think these 128 billion were given to developing countries, but two-thirds were awarded to companies in the North, only benefiting the North's economy. Aid doesn't work as well as it could because it is not delivered in the way it should be delivered”.

### Eurodad's report critical of World Bank aid's procurement practices

The Eurodad study also showed that half of the contract value in World Bank-funded projects in the last decade went to firms from donor countries, with the share increasing with the size of the contract. In 2008, 67%of all World Bank-financed contracts went to firms from just 10 countries as “...a consequence of World Bank procurement practices”. The study explains that most recipient countries are pressured to allow transnational companies to bid for contracts. Aid is given if the market opens up to international competition, benefiting western companies. Eurodad calls for “smart procurement” and preferential access for local or regional companies to be awarded aid contracts. Smart procurement also means “...imposing conditions on contractors that ensures that aid contributes to sustainable development”.

## United Nations (UN)

### Donors provided nearly US$ 375 million to UN’s emergency relief fund

According to the UN News, donors pledged nearly US$ 375 million (€ 289 million) to the United Nations emergency relief fund. This money is meant to ensure that humanitarian workers can quickly begin saving lives whenever a humanitarian crisis strikes anywhere in the world. The Central Emergency Response Fund (CERF) has disbursed more than US$ 2 billion (€ 1.5 billion) in assistance to different stricken areas since it was launched in the year 2006, making it the world's largest source of humanitarian funding.

### UN calls for better land governance to fight corruption and “land grabs”

Countries must increase governance and transparency in land use to fight corruption, the United Nations' Food Agency said in a report following the UN’s talks on land governance in Rome. The joint working paper by the U.N.'s Food and Agriculture Organisation (FAO) and global graft watchdog Transparency International found corruption in the agriculture sector varies from small fraud to high-level abuses of government power. Governments should expect speculation and monitor concentration of ownership when land rights are transferred to investors to “develop” farmland. A UN special rapporteur on the right to food Olivier De Schutter spoke for The Guardian earlier this year: “We must escape the mental cage that sees large-scale investments as the only way to develop agriculture and to ensure stability of supply for buyers”. The recent surge in food prices has prompted both investors and governments to focus on agriculture after decades of neglect, which eventually brought the attention on land deals in developing countries. A recent report by Oxfam identified 227 hectares of land – an area the size of northwest Europe – as having being reportedly sold, leased or licensed, largely in Africa and mostly to international investors in thousands of secretive deals since 2001 – i.e., considerably more than the World Bank’s estimate of 56 hectares.

### UN Population Fund warns that high fertility impedes economic development

The UN Population Fund (UNPF) has warned that continued high fertility in sub-Saharan Africa and southern Asia is impeding economic development and perpetuating poverty in those regions. With an estimated 215 million women seeking family planning each year, but unable to gain access to it, family planning would need to be considered a priority among the local policy-makers. In its annual state of world population report, the UNPF called for health and education programs to improve this situation and enhance the development of those regions. The report was issued ahead of the human population’ predicted growth to 7 billion at the end of October this year.

### UN population chief says family planning opportunities missed because of HIV/AIDS focus

The Guardian reported on the interview recently given by Babatunde Osotimehin, the executive director of the UN Population Fund, related to the release of UNPF’s state of world population report. Mr Osotimehin said that the international community may have “made a mistake” with the intensity of its focus on the global HIV-AIDS epidemic. Because of this effort, the ground was lost on family planning issues as a result. He added that “…efforts to expand family planning services in the developing world stalled for a decade, while global health organizations turned their energies to fighting HIV/AIDS”.

### Slow start to global movement against non-communicable diseases

Globe and Mail, Centre for Global Development, the World Health Organization's Press Office and many others have been following and reporting from a two-day United Nations summit on the prevention and control of non-communicable diseases (NCD). The very first gathering of such kind took place in New York in September – and according to *Globe and Mail*, it ended «...with a whimper rather than a bang». The heads of state and government leaders, senior ministers and experts who attended the meeting, all took some very modest steps to get the movement started - but according to the observers, far too few (and too small) were taken to address the urgency and magnitude of the challenge the world now confronts. The main risk factors for NCDs today are unhealthy diet, physical inactivity, tobacco use and alcohol abuse. Interventions against them could counter the NCD pandemics for a relatively modest price in comparison to the expected benefits. However, the attendees of the New York summit did not seem interested in this bargain, all of them withstanding an intense fiscal consolidation mode under the current economic climate. Ms Glassman from the Centre for Global Development suggests that “... the main outcome ... is homework for the WHO, ... (now) assigned to lead the global response to NCD, develop a monitoring and evaluation framework, provide technical assistance and track progress towards global targets – (which is) yet another unfunded mandate while WHO is in the midst of a major reform precipitated by funding cuts itself”.

## UN-AIDS

### Three US presidents mark World AIDS Day amidst funding crisis

Thirty years after AIDS surfaced, Mr Barack Obama, declared “the beginning of the end” of the disease, which is primarily due to dramatic positive results achieved by antiretroviral drugs. Mr Obama was joined on World Aids Day by two of his predecessors who also contributed to the progress - Mr Bill Clinton and Mr George W. Bush. However, this declaration came in parallel with the decision by the Global Fund to Fight AIDS, TB and Malaria to call off its latest funding round, as countries around the world are slashing their aid budgets amid global financial strife.

### HIV/AIDS: Delayed Global Fund money a sign of economic times

*IRIN* reported that The Global Fund to Fight AIDS, TB and Malaria “…has more than halved the estimated amount of money available in its next round of funding, the disbursement of which has been delayed until 2013, due to the world economic crisis”. The delay in Round 11 funding was announced at the Fund's latest board meeting in September, pushing the application deadline back to at least March 2012. The size of the available support has also decreased - to US$ 800 million (€ 617 million), which is less than a half of the US$ 1.5 billion (€ 1.6 billion) projected for the round as of mid-2011, according to Mr Christoph Benn, director of the Fund's external relations and partnerships.

### UNAIDS chief endorses financial transaction tax as a way to bridge funding gap

Reuters reported recently that the Head of UNAIDS, Mr Michael Sidibe, has warned of a setback in the fight against HIV/AIDS following the funding crunch of the Global Fund. Mr Sidibe “…called for a financial transaction tax and other taxes to finance the ongoing AIDS response instead”. A linked article in the leading scientific journal *Nature* warned that donor cutbacks are threatening gains in HIV treatment. Meanwhile, UNAIDS reported the highest number of HIV infections ever in Middle East and North Africa in the year 2010, but called recent progress “promising”.

### A developing country-based alliance to develop child-friendly AIDS drugs

The Drugs for Neglected Diseases Initiative (DNDi), an international non-profit drug research and development organization and scientific alliance in which developing countries have the key role, has embarked on producing paediatric antiretroviral (ARV) drugs. This is a welcome initiative because this area is of little interest to large pharmaceutical companies, given that mother-to-child transmission of HIV has practically been eliminated in the industrialised world.

### Global campaign launched against Abbott over monopoly on a key AIDS drug

The Guardian reported that multinational drug company Abbott is targeted by health campaigners in several countries. They are trying to break its monopoly on AIDS drug Kaletra (also known as Aluvia). This is a combination of two drugs, lopinavir and ritonavir, the latter partly developed with funding from the US government, as the campaigners point out. It is becoming increasingly important in the developing world, as the resistance to first-line drugs grows, but it is under patent to Abbott and disproportionately expensive. Basic AIDS drugs prices have been reduced from US$ 10 000 (€ 7700) per patient per year to about US$ 100 (€ 77) through generic competition, but Abbott did not allow very cheap generic copies to be made. Abbott charges the poorest countries in the world US$ 400 (€ 309), while middle-income developing countries have to pay between US$ 1000 (€ 770) and US$ 4000 (€ 3090). Public Citizen in the USA is leading the charge, but campaigners in Brazil, India, Vietnam, Indonesia, Colombia, Thailand, the Netherlands and elsewhere are all taking action - mostly by challenging Abbott's monopoly in their own legal systems.

## UNICEF

### UNICEF chief urges action over Sahel food crisis

Just ahead of Christmas, the Head of the United Nations Children's Fund (UNICEF) Mr Anthony Lake urged international action to prevent one million children in the Sahel region of West and Central Africa from becoming severely malnourished. He said that “…the region is vast, the challenge is great and the window is closing”, and added “…to prevent a wide-scale emergency in the Sahel, UNICEF and our partners in this effort must begin at once to fill the pipeline with life-sustaining supplies to the region before it is too late”. Malnutrition among children is already prevalent in the Sahel region, and the urgency is prompted by the ‘lean season’, when food usually runs out due to inadequate rain or poor harvests. This period can start as early as March. Mr Lake added that “…specially developed ready-to-use therapeutic foods are the best way to treat severe acute malnutrition among children under five so they have a chance to survive and recover”, he added, concluding that “…the children in the Sahel are not mere statistics by which we may measure the magnitude of a potential humanitarian disaster. They are individual girls and boys, and each has the right to survive, to thrive and to contribute to their societies. We must not fail them”. UNICEF appealed for nearly US$ 66 million (€ 51 million) to respond to the crisis.

### UNICEF and WHO work together to prevent further mortality in the Horn of Africa

Since July this year, when famine was declared in parts of southern Somalia, UNICEF and its partners have been working hard to prevent a second, potentially more devastating wave of deaths from disease, within a context of ongoing conflicts in the region. In Mogadishu, a UNICEF and WHO-supported measles vaccination campaign began in November for 750 000 children who are 6 months to 15 years old. It comes after further 1 million children have already been vaccinated against measles in Somalia during the months after the famine struck parts of the country.

### An innovative partnership between Pampers and UNICEF on track to reduce neonatal tetanus

An innovative partnership between UNICEF and Procter & Gamble's largest brand, “Pampers”, has proven a real success in a fight against neonatal tetanus – a disease that is estimated to kill a baby or a mother somewhere in the world every 10 minutes. In 2008, Procter & Gamble vowed to contribute a portion of the revenue from each pack of Pampers during the fourth quarter toward a vaccine against neonatal tetanus. This started as a small pilot program in Western Europe, but since then enthusiasm from the consumers has been so strong, and the financial support to this initiative so substantial, that the disease may be eliminated by 2015. Although Pampers became one of UNICEF's largest corporate donors over the past few years, the campaign has in fact delivered year-on-year growth for Pampers sales – even in its toughest markets. This success is a new model for “cause-related marketing”.

### UNICEF dismiss concerns over polio outbreak in Madagascar

According to BBC, the United Nation children's fund (UNICEF) has denied that there has been a polio outbreak in Madagascar. UNICEF said that the mistaken concern over an outbreak of wild poliovirus came after its office in Madagascar had issued a statement that vaccine-derived poliovirus had been detected in three health children amid an immunization campaign. This in no way implied a new outbreak of polio. In fact, the last case of polio was detected on Madagascar in 1997. According to UNICEF, “…the release may have led to a misunderstanding that there is an outbreak of wild poliovirus in Madagascar”.

### UNICEF launches Web site on global polio immunization

UNICEF announced this fall that it has launched a new Web site – PolioInfo – “…to support and strengthen communication efforts in all the polio priority countries”. The new Web site should make this critical social data easier to access. The Web site is linked to the official Web site of the “Global Polio Eradication Initiative” (GPEI), which focuses on the epidemiological and logistical aspects of polio eradication. UNICEF concluded that “…the two websites will work in harmony to provide a complete array of information to experts and community members”.

## World Health Organization (WHO)

### Margaret Chan proposes “WHO priority setting” to guide the reforms

WHO’s Executive Board met in October to review progress made on the reforms that were proposed earlier this year. One of the key documents presented to the Executive Board was a report on plans for priority-setting amongst the WHO’s 213 projects which are currently run by its 8 divisions and 15 regional and special offices. In an era of serious resource shortage at the organization and its decreasing importance in the global health arena, a profound reform based on transparent priority setting process and reliance on comparative advantages seems like a good plan, at least in principle. The WHO’s strategy document listed five “core areas” of work: (i) health development; (ii) health security; (iii) health systems; (iv) evidence; and (v) convening. The document implies that they are what WHO “does best”, and they “…distinguish WHO from organizations whose prime function is to manage and disburse loans and grants as their main lines of business”, and “…from institutions that develop knowledge without necessarily being responsible for its application”.

### WHO define “flagships that reflect global concerns” within their five priority areas

Within the five core areas that WHO proposed to prioritize through their “priority setting”, as explained in the previous news item, they went further and defined “flagships… that reflect global concerns”. These should be more specific areas of focus within the main priority areas. They were listed as follows: (i) communicable and non-communicable disease; (ii) health systems; (iii) equitable access; (iv) support to country achievement of MDG.

### Analysts point to lack of transparency in WHO’s new priority setting

A number of reporters, analysts and commentators were quick to point to an apparent lack of transparency in WHO’s newly proposed “priority setting”. Amanda Glassman from the Centre for Global Development questioned the transparency of this process and the choices that were being made. There seemed to be no justification in the document why are these “flagships” chosen and how exactly are, eg, infectious diseases, health systems, supporting individual countries, or equity “reflect global concerns”. Also, some questioned how prioritizing “communicable and non-communicable diseases” is “more focused”, and how does it represent WHO’s comparative advantage at this point in time. According to Ms Glassman, “…the report does not offer an evaluation of “what is done best” nor does it explain the mysterious, somewhat passive-aggressive reference to other organizations and its implications for WHO’s comparative advantage”. The reference to “…organizations whose prime function is to manage and disburse loans and grants” probably implies donor organizations such as The Global Fund, GAVI and The Gates Foundation, while a reference to “…institutions that develop knowledge without necessarily being responsible for its application” probably refers to academic institutions. However, only several years ago WHO was still the main policy developer in global health, the main distributer of the funding, and its staff was being considered to have technical supremacy in global health issues. Nowadays, they are being overshadowed in all these areas by the large new donor initiatives and the academic community. This is an entirely uncharted territory for the WHO leadership and it will be interesting to see how will they respond to these challenges and whether they are able to indeed conduct a reform that would return some of the relevance that has been lost to other organizations over the past decade. Still, most of the analysts agree that historic role of the WHO in combating communicable diseases is commendable, while their appreciation of the growing non-communicable disease burden should also be welcomed.

### WHO’s World Health Report for 2012 will focus on health research

The annual World Health Report, first published in 1995, is WHO's flagship publication which includes an assessment of an important global health topic and provides a world-wide assessment. In 2012 the theme will be “No Health Without Research”, thus becoming the first World Health Report to highlight and analyze the impact of health research. The theme should meet WHO's core function of “stimulating the generation, translation and dissemination of valuable knowledge” and it will be assembled by Dr Tikki Pang, Director of Research Policy and Cooperation at the WHO. In a blog posted in January this year, Dr Pang had set a tone to this forthcoming report by proposing that “…ministers of health in developing countries must strive to strengthen health research in their countries by addressing several key questions, which will be the focus of the World Health Report 2012: How should research priorities be set? What human and institutional capacities are needed? How to ensure ethical and good behaviour? How to promote transparency and accountability? How should knowledge be translated into action? What is the best way to coordinate research among the many performing it, and when so many health challenges involve sectors beyond health?” The open-access journal *PLoS Medicine* will publish a special collection of papers related to this theme.

### An article prompted WHO to disclose its funding sources

An article published by ForeignAffairs.com by Sonia Shah, entitled “How private companies are transforming the global public health agenda”, prompted a strong response from Ms Christy Feig, the Director of Communications at the WHO. Ms Feig accused Ms Shah of making a number of erroneous statements about how the WHO is funded, primarily the one which claimed that “…voluntary contributions from private interests and others now bankroll four out of every five dollars of the WHO's budget”. Ms Feig disclosed that 80% of WHO's budget is actually coming from world’s governments, and that within the two-year budget period 2010-2011 precisely “…53% of the voluntary contributions came directly from governments that chose to go beyond what their annual dues require”. Further 21% of voluntary contributions came from other UN bodies (such as UNICEF, UNDP and UNAIDS) and other multilateral bodies (such as the GAVI), and another 18% from large donor foundations (such as the Bill & Melinda Gates Foundation, the UN Foundation, and the Rockefeller Foundation). Of the remainder, 7% came from nongovernmental organizations, which were dominated by funding from Rotary International for work on polio eradication.

